# Immune-mediated competition in rodent malaria is most likely caused by induced changes in innate immune clearance of merozoites

**DOI:** 10.1186/1475-2875-13-S1-P78

**Published:** 2014-09-22

**Authors:** Jayanthi Santhanam, Lars Raberg, Andrew Read, Nick Savill

**Affiliations:** 1 University of Edinburgh, Edinburgh, UK; 2 Lund University, Lund, Sweden; 3 The Pennsylvania State University, Pennsylvania, USA

## 

Malaria infections often consist of more than one strain of the same parasitic species. Understanding the within-host competition between these various strains is essential to understanding the evolution and epidemiology of drug resistance in malarial infections. The infection process and the competition between strains involve complicated biological processes that are explained by various hypotheses. Mathematical models tested against experimental data provide quantitative measures to compare these hypotheses and enable us to discern the actual biological processes that contribute to the observed dynamics. We use a group of models against experimental data on rodent malaria to test various hypotheses. Such quantitative measures, in understanding rodent malaria, can be considered as a step towards understanding within-host parasite dynamics. Our work presented here demonstrates how confronting mathematical models with data allows the discovery of subtle and novel interactions between hosts and parasites that would be impractical to do in an experiment and allows the rejection of hypotheses that are incorrect. It is our contention that understanding the forces controlling within-host parasite dynamics in well-defined experimental model is a necessary step towards understanding these features in natural infections.

